# The impact of apolipoprotein E, type ∊4 allele on Alzheimer’s disease pathological biomarkers: a comprehensive post-mortem pilot-analysis

**DOI:** 10.1371/journal.pone.0303486

**Published:** 2025-02-06

**Authors:** Ziyu Wan, Tao Ma

**Affiliations:** 1 Department of Internal Medicine, Gerontology and Geriatric Medicine, Wake Forest University School of Medicine, Winston-Salem, North Carolina, USA; 2 Department of Translational Neuroscience, Wake Forest University School of Medicine, Winston-Salem, North Carolina, USA; Institute of Neurophysiopathology, FRANCE

## Abstract

The apolipoprotein E type ∊4 allele (ApoE4) is known as the strongest genetic risk factor for Alzheimer’s Disease (AD). Meanwhile, many aspects of its impact on AD pathology remain underexplored. This study conducts a systematic data analysisof donor data from the Seattle Alzheimer’s Disease Brain Cell Atlas. Our investigation delves into the intricate interplay between identified biomarkers and their correlation with ApoE4 across all severities of AD. Employing Pearson R correlation, and one-way and two-way ANOVA tests, we elucidate the pathological changes in biomarkers and the altering effects of ApoE4. Remarkably, the phosphorylation of tau observed in neurofibrillary tangles (NFTs) marked by the AT8 antibody, emerges as the most correlated factor with other pathological biomarkers. This correlation is mediated by both tau and amyloid pathology, suggesting a higher hierarchical role in determining AD pathological effects than other biomarkers. However, non-ApoE4 carriers exhibit a more significant correlation with disease progression severity compared to ApoE4 carriers, though ApoE4 carriers demonstrate significance in exacerbating the effect of accumulating phosphorylated tau and amyloid plaques assessed by AT8 and 6E10 antibodies. Furthermore, our analysis does not observe dramatic neuronal changes in grey matter across the span of AD pathology. Glia activation, measured by Iba1 and GFAP, demonstrates an amyloid-specific correlation. This research marks the first human post-mortem analysis providing a comprehensive examination of prevailing AD biomarkers and their interconnectedness with pathology and ApoE4 genetic factor. Limitations in the study are acknowledged, underscoring the need for further exploration and refinement in future research endeavors.

## Introduction

Apolipoprotein E, type ∊4 allele (ApoE4) is one of the most significant genetic risk factors of Alzheimer’s disease (AD) and is associated with both familial (early-onset) and sporadic AD [1]. Multiple studies demonstrated the robust correlation between the number of ApoE4 alleles and the risk of developing AD, with a single allele elevate the risk to two to four times and having two alleles escalating it eight to twelve times more [2,3]. Additionally, ApoE4could significantly lower AD age [2,4]. Besides AD, ApoE4 has been implicated in increasing the risk of cardiovascular disease, stroke, and other neurodegenerative disorders [2].

Within the central nervous system (CNS), ApoE is predominantly produced in the astrocytes and microglia upon activation by injuries and to a lesser extent, in neurons under stress conditions [5–7]. ApoE has three main isoforms: ApoE2, ApoE3, and ApoE4, among which, ApoE3 and ApoE4 are notably distinct in their impact on AD pathology. ApoE3 is generally considered neutral with respect to AD risk, while ApoE4 is strongly associated with an increased risk of developing the disease. In contrast, ApoE2, though less common, is considered protective against AD [2,5,8,9]. Ample research has shown that ApoE4 carriage is associated with numerous AD-related pathologies such as amyloid beta (Aβ) aggregation, tau hyperphosphorylation, and neuroinflammation. ApoE4 may also exacerbate accumulations of neurofibrillary tangles (NFTs) and amyloid plaques, two cardinal AD lesions [10,11].

Interactions between Aβ pathology and ApoE4 are one of the most defined parts of AD pathogenesis. Aβ peptides (mainly Aβ40 and Aβ42) are generated from the Amyloid precursor protein (APP) through cleavage by the enzymes β-secretase and γ-secretase [12–14]. ApoE4 facilitates Aβ deposition, especially Aβ42 [15,16], while apoE2 appears to reduce the accumulation of Aβ.

Hyperphosphorylated microtubule-associated protein tau constitutes a significant portion of neurofibrillary tangles (NFTs), a hallmark brain pathology of AD [17]. Similar to its impact on amyloid pathology, ApoE4 may exacerbate the tauopathy based on studies from both mouse models and humans [18–20]. The correlation between ApoE4 and tau phosphorylation could be independent of amyloid pathology that the NFTs are found to be associated with ApoE4 regardless of amyloid plaques presence [21]. Moreover, apoE2 doesn’t appear to elicit a protective effect on tauopathy as in the case of the Aβ depositions [10,22], and may exacerbate tauopathy to some extent [23]. In addition, plasma and cerebrospinal fluid (CSF) phosphorylated tau (p-tau), particularly its isoform p-tau181 and p-tau217, are recognized as potential biomarker for early onset AD in preclinical settings and the level of p-tau has been associated with the intensity of tauopathy [24–29].

The initiation and progression of AD are linked to neuroinflammation, eliciting responses from both microglia and astrocytes, and recent studies indicated that ApoE plays a crucial role in modulating inflammation in AD [11,30]. TREM2, triggering receptor expressed on myeloid cells, shows high affinity with apoE and significantly modulate microglia responses. Additionally, ApoE4 may induce microglia homeostatic imbalance,potentially due to its reduced lipidation and reduced affinity to TREM2 [30]. However, a recent study using mouse models demonstrates that TREM2 suppression does not protect against tau pathology in the presence of ApoE4 [31].

There are notable gaps in understanding the precise contributions of ApoE4 allele to AD pathology. While substantial research has utilized in vivo imaging and animal models, few comprehensive studies have examined ApoE4 and assorted AD biomarkers within human neurological tissue. This paucity is impactful, as post-mortem assessments can unveil disease end-stage qualities potentially distinct from earlier phases or non-human models.

Our study aims to address these gaps by conducting a systematic analysis of the post-mortem profiling of AD biomarkers considering ApoE4 carriage status. We focused on the middle temporal gyrus (MTG) of the cortex, which was sampled and quantified by the SEA-AD organization for Alzheimer’s pathophysiology. We aim to 1. Investigate relationships between ApoE4 and pivotal AD biomarkers, including p-tau, Aβ deposits, neuronal loss, and glial reactivity; 2. Assess the potential of these biomarkers to discriminate ApoE4 carriers from non-carriers in AD brains; 3. Evaluate how ApoE4 presence influences the severity and progression patterns of these markers across AD stages. Through this comprehensive analysis, we aim to uncover the associations between significant biomarkers, genetic risk factors, cognitive status, and sex differences in AD. In turn, this may inform the development of targeted treatments and improve diagnostic accuracy, especially for individuals facing genetic AD predisposition due to ApoE4 allele carriage.

## Result

We examined ApoE4 status in correlation with AT8 and 6e10 antibodies; Ionized calcium-binding adaptor molecule 1 (Iba1) microglia and glial fibrillary acidic protein (GFAP) activation; Neuronal loss; p-tau, Aβ, and their ratios, of which were sampled from the MTG of the cortex. In addition, we evaluated their roles in cognitive examinations and gender differences. P-Tau and Aβ 42 levels were measured under both Radioimmunoprecipitation Assay (Ripa) and Guanidine Hydrochloride (Guhcl) protein precipitation.

Details of the groups and categories used in the analysis are included in Table 1.

**Table 1:** Distribution of participants based on ApoE4 and cognitive status under female and male cohorts.

Female (# of participants)			Total
Cognitive Status_/ApoE4 Status_	No dementia –Nodem	Dementia –Dem	
ApoE4 Negative –N	NNodem [19]	NDem [16]	35
ApoE4 Positive –Y	YNodem [5]	YDem [11]	16
Total	24	27	51

Male (# of participants)			
Cognitive Status/_ApoE4 Status_	No dementia –Nodem	Dementia –Dem	
ApoE4 Negative –N	NNodem [14]	NDem [10]	24
ApoE4 Positive –Y	YNodem[4]	YDem [5]	9
Total	18	15	33

### p-Tau/ Aβ aggregation

**Fig 1:**
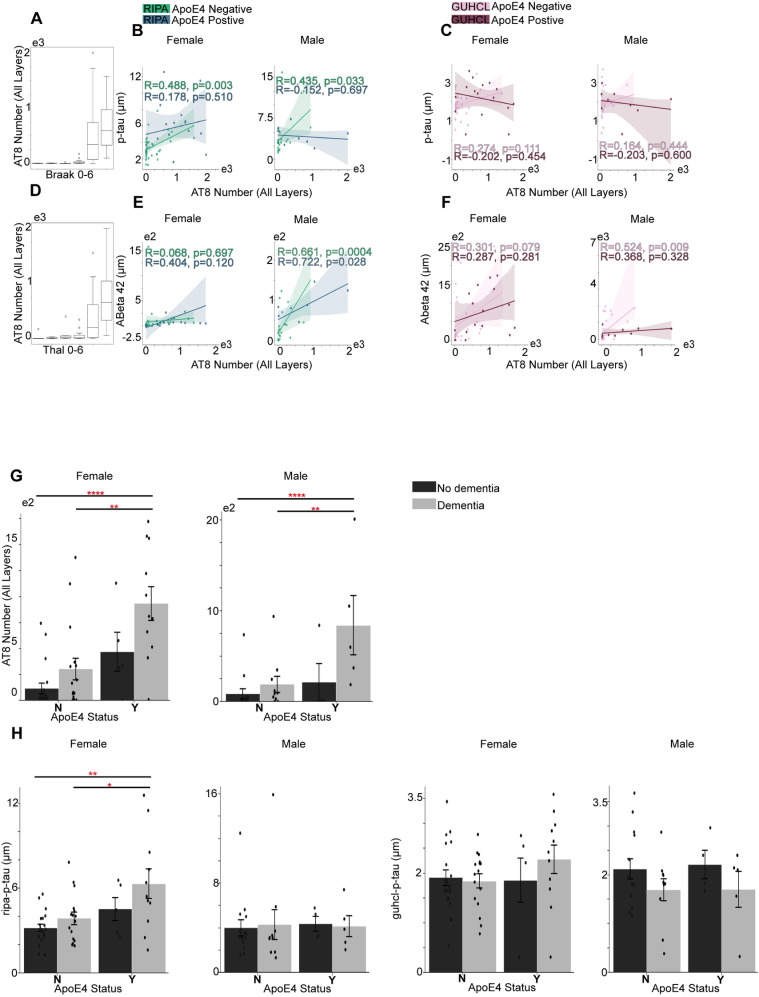
Grey matter AT8 positive cells over the progression of Tau phosphorylation and Aβ aggregation, and their associations with ApoE4 and cognitive status. (A) Total number of AT8’s progression in correlation with Braak stages. (B) The association between Ripa immunoassay of p-Tau level and the total number of AT8 under different sex groups. (C) The association between Guhcl protein precipitation of p-Tau level and the total number of AT8 in different sex groups. (D) Total number of AT8’s progression in correlation with Thal stages. (E) The association between Ripa immunoassay of Aβ42 level and the total number of AT8 in different sex groups. (F) The association between Guhcl protein precipitation of Aβ42 level and the total number of AT8 in different sex groups. (G) ApoE4 and cognitive status influence on AT8 cells aggregation under different sex groups. (H) ApoE4and cognitive status influences on p-Tau levels (Ripa and Guhcl) under different sex groups. Pearson’s R correlation was conducted B-C, and E-F. Two-way ANOVA and Tukey’s HSD post-hoc analysis was used for G and H. Data represent mean ±  SEM. * p <  0.05; **p <  0.01; ***p <  0.001; ****p <  0.0001.

**Fig 2:**
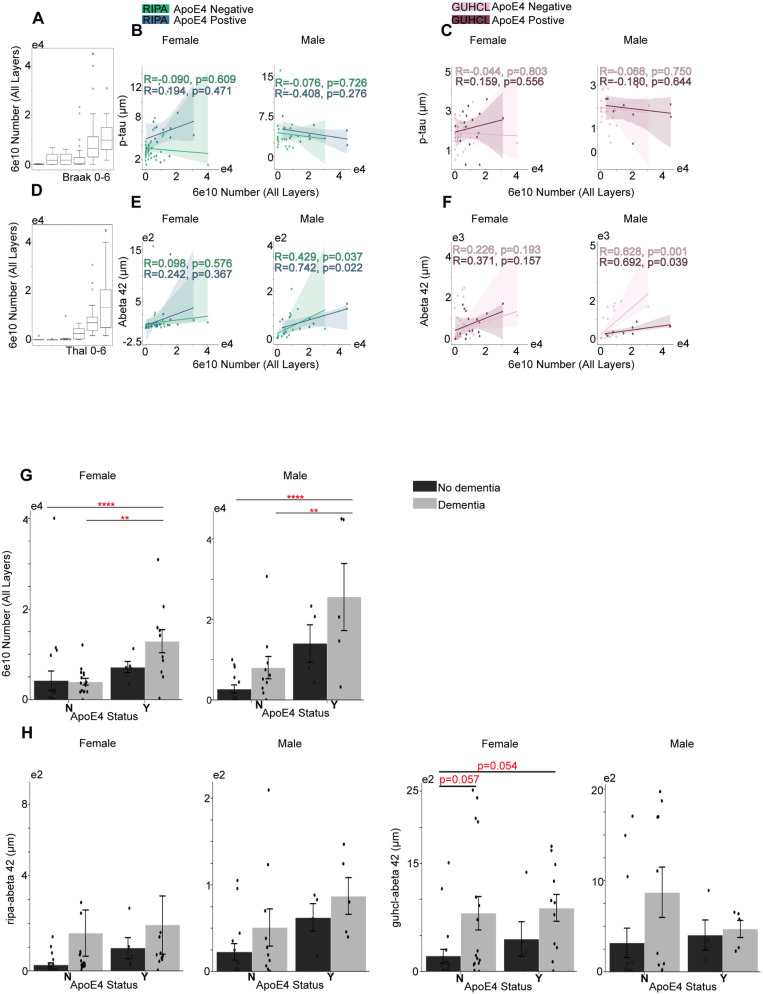
Grey matter 6e10 positive cells over the progression of Tau phosphorylation and Aβ aggregation, and their associations with ApoE4 and cognitive status. (A) Total number of 6e10’s progression in correlation with Braak stages. (B) The association between Ripa immunoassay of p-Tau level and the total number of 6e10 under different sex groups. (C) The association between Guhcl protein precipitation of p-Tau level and the total number of 6e10 different sex groups. (D) Total number of 6e10’s progression in correlation with Thal stages. (E) The association between Ripa immunoassay of Aβ42 level and the total number of 6e10 in different sex groups. (F) The association between Guhcl protein precipitation of Aβ 42 level and the total number of 6e10 in different sex groups. (G) ApoE4 and Cognitive status influences on 6e10 cells aggregation under different sex groups. (H) ApoE4 and Cognitive status influences on Aβ42 levels (Ripa and Guhcl) under different sex groups. Pearson’s R correlation was conducted B-C, and E-F. Two-way ANOVA and Tukey’s HSD post-hoc analysis was used for G and H. Data represent mean ±  SEM. * p <  0.05; **p <  0.01; ***p <  0.001; ****p <  0.0001.

ApoE4 negative group, AT8 positive cells showed moderate correlation in identifying the phosphorylated tau (p-Tau) in both females and males under Ripa (r = 488, r = 0.435) but not Guhcl precipitation (Fig 1B and 1C). Interestingly, AT8 positive cell numbers revealed a strong positive correlation in recognizing amyloid plaques (Aβ42) in male ApoE4negative group under both Ripa and Guhcl assays, r = 0.661, r = 0.524 respectively. In addition, the ApoE4 positive group of males exhibited an even stronger correlation of AT8 and Aβ42 level, r = 0.722. In contrast, the numbers of 6e10 exhibit a strong positive correlation only with Aβ42 level in male groups, regardless of ApoE4 status, showing its specificity of identifying amyloid plaques [32]. The male ApoE4 positive groups showed a higher correlation than negative groups in both Ripa and Guhcl measurements with r = 0.742 and 0.692 (Fig 2E and 2F) [33].

Although Pearson correlation tests reveals more significant correlations in ApoE4-negative group than ApoE4-positive group in identifying p-tau, contradicting previous findings [8,18], two-way ANOVA revealed ApoE4 status independently affecting the accumulation of AT8 numbers for both female and male cohorts, F (1,47) =  18.96, p <  0.001 and F (1,29) =  7.65, p =  0.010, respectively [11,20,30]. Cognitive status revealed a main effect under female group only, F (1,47) = 5.55, p =  0.023. The following Tukey’s HSD tests were conducted to investigate the possible interactive effect. For ApoE4’s effect in female group, carriers (Y) had significantly higher levels of AT8 antibodies than non-carriers (N), with a mean difference of 585.76, p <  0.001. Male group also exhibited higher AT8 in Y than N with a mean difference of 433.31, p =  0.009. Regarding cognitive status effects on female AT8 cell numbers, individuals with dementia (Dem) showed significantly higher levels of AT8 antibodies compared to those without dementia (Nodem), with a mean difference of 368.55, p =  0.006. Moreover, we found that NNodem significantly differ from the YDem group, and NDem significantly differ from the YDem group in both female and male cohorts (Fig 1G). YDem in female is significantly higher than the NNodem group with a mean difference of 815.48, p <  0.001, and higher than NDem group of 631.14 units, p =  0.008. Male YDem is also significantly higher than NNodem group with a mean difference of 755.67, p =  0.002, and higher than NDem group of 651.70, p =  0.015. Two-way ANOVA analysis on 6e10 antibodies also showed ApoE4 independent effects in both female and male, with F (1,47) =  8.81, p =  0.005 in female and F (1,29) =  15.59, p <  0.001 in male. Cognitive status only showed significant effect within male cohort, F (1,29) =  4.61, p =  0.040. Notably, Tukey’s HSD test also exhibited significant pairwise differences between the NNodem - YDem and NDem - YDem groups regarding 6e10 antibodies as AT8 for both female and male, with YDem contained the highest mean of 6e10 cell numbers (Fig 2G). Female YDem and NNodem had a mean difference of 8685.22, p =  0.014, male groups had a mean difference of 22898.43, p <  0.001. Female YDem and NDem revealed a mean difference of 8899.32, p = 0.016, while male groups revealed a mean difference of 17537.80, p =  0.010.

ApoE4 status only shows a significant independent effect in the female group on Ripa-pTau level, F (1,47) =  9.97, p =  0.003. Later Tukey’s HSD revealed significant pairwise differences between female-specific NNodem - YDem, NDem – YDem groups, with the YDem group having the highest mean (Fig 1H). YDem had significantly higher means of 3.11 than NNodem, p =  0.001, and significantly higher means of 2.44 than NDem, p =  0.021, while the male groups exhibited no significance at all with p =  1.00. In addition, no significance was found under Guhcl-pTau measurements with the influence of ApoE4 or cognitive status. The sex specificity effect was observed for the first time that ApoE4 presence significantly aggregates Ripa-pTau level but not in the male cohort. However, male cohort two-way ANOVA shows ApoE4 almost had a significant effect on Ripa- Aβ42 that F (1,29) =  3.89, p =  0.058. Despite the insignificant result shown from two-way ANOVA, Tukey HSD revealed a significant difference in Ripa- Aβ42 level between ApoE4 Y and N, with Y showing higher levels by an average of 41.74 units, p = 0.04. Interestingly, cognitive status presented a significant main effect on female guhcl- Aβ42 level, with F (1,47) =  8.09, p = 0.007. However, Tukey HSD shows no further significance over pairwise differences (Fig 2H). This suggests that the ApoE4 effect on Aβ 42 peptides level shown in post-mortem samples were not as significant as stated [15,16].

### Microglia/ astrocyte activation

Glia activation, involving both microglia and astrocytes, is recognized as another hallmark of AD onset and progression triggered by inflammation of synapses and neurons [10,34].

**Fig 3:**
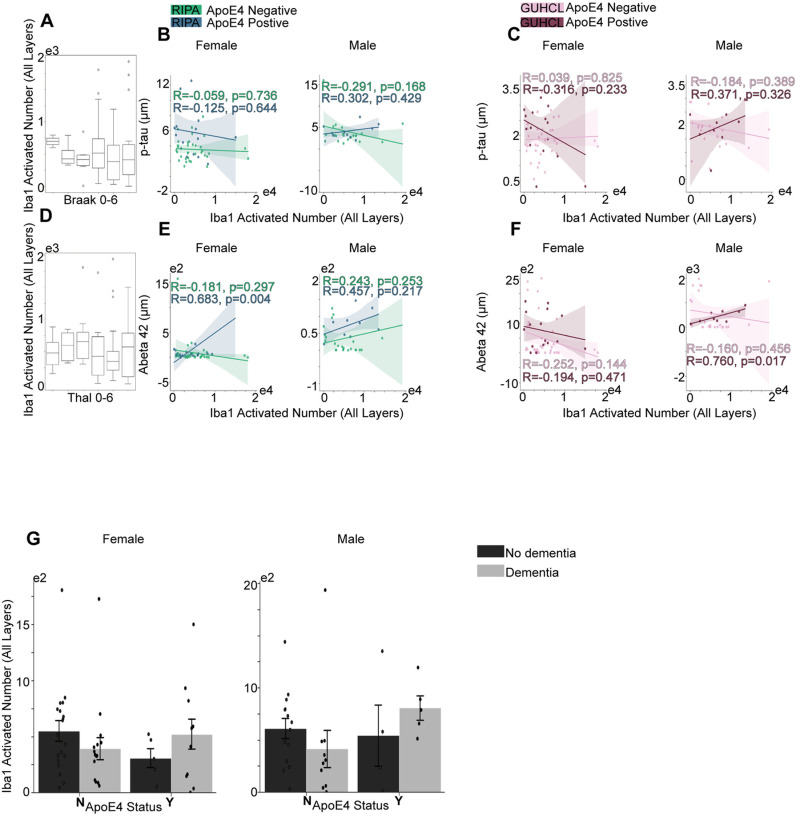
Grey matter Iba1 activation over the progression of Tau phosphorylation and Aβ aggregation, and their associations with ApoE4 and cognitive status. (A) Total number of Iba1’s progression in correlation with Braak stages. (B) The association between Ripa immunoassay of p-tau level and the total number of Iba1 under different sex groups. (C) The association between Guhcl protein precipitation of p-Tau level and the total number of Iba1 different sex groups. (D) Total number of Iba1’s progression in correlation with Thal stages. (E) The association between Ripa immunoassay of Aβ42 level and the total number of Iba1 in different sex groups. (F) The association between Guhcl protein precipitation of Aβ42 level and the total number of Iba1 in different sex groups. (G) ApoE4 and Cognitive status influences on Iba1 cells aggregation under different sex groups. Pearson’s R correlation was conducted B-C, and E-F. Two-way ANOVA and Tukey’s HSD post-hoc analysis was used for G. Data represent mean ±  SEM. * p <  0.05; **p <  0.01; ***p <  0.001; ****p <  0.0001.

**Fig 4:**
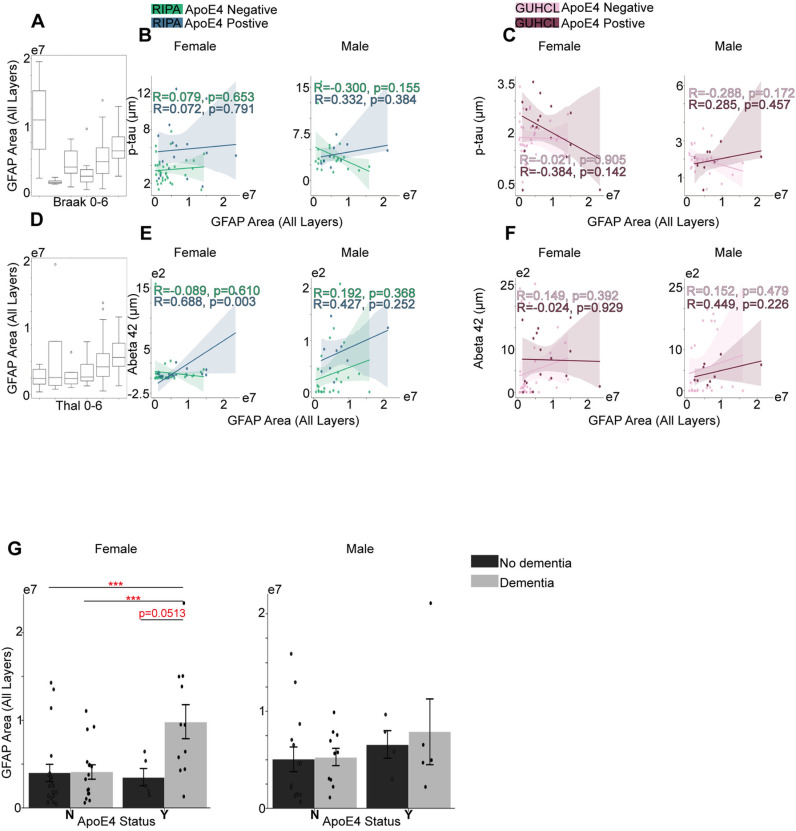
Grey matter GFAP activation over progression of Tau phosphorylation and A β aggregation, and their associations with ApoE4 and cognitive status. (A) Total number of GFAP’s progression in correlation with Braak stages. (B) The association between Ripa immunoassay of p-tau level μm and the total number of GFAP under different sex groups. (C) The association between Guhcl protein precipitation of p-Tau level and the total number of GFAP different sex groups. (D) Total number of GFAP’s progression in correlation with Thal stages. (E) The association between Ripa immunoassay of Aβ42 level μm and the total number of GFAP in different sex groups. (F) The association between Guhcl protein precipitation of Aβ42 level and the total number of GFAP in different sex groups. (G) ApoE4 and Cognitive status influences on GFAP cell aggregation under different sex groups. Pearson’s R correlation was conducted B-C, and E-F. Two-way ANOVA and Tukey’s HSD post-hoc analysis was used for G. Data represent mean ±  SEM. * p <  0.05; **p <  0.01; ***p <  0.001; ****p <  0.0001.

Based on the Pearson correlation, we did not observe significant correlation between either the Iba1 activation or GFAP activation and the level of p-Tau regardless of ApoE4 status or gender differences for both Ripa and Guhcl measurements (Figs 3B,3C, 4B and 4C). In contrast, we noticed a strong positive correlation between Iba1 activation and the Aβ42 aggregation in female ApoE4 positive carriers, with a r =  0.683, p = 0.004. In addition, under Guhcl measurement, Iba1 also exhibited strong positive correlation with Aβ42 as well in the male ApoE4 positive group, r =  0.760, p =  0.017 (Fig 3E and 3F). Moreover, a similar significant correlation was observed in female positive group between the GFAP activation and Aβ42 level using Ripa immunoassay, r =  0.688, p =  0.003 (Fig 4E and 4F). This shows a specific Aβ aggregation correlation with the immunoreactivities of microglia and astrocyte cells.

We further investigate the performed two-way ANOVA analysis on Iba1 and GFAP activation over ApoE4 and cognitive impact. ApoE4 and cognitive status didn’t exhibit any significant influence on Iba1 activation, F (1,47) =  0.008, p = 0.930, and F (1,47) =  0.216, p =  644, respectively in female cohort; F (1, 29) =  0.919, p =  0.346, F (1,29) =  0.183, p =  0.672, respectively in male cohort (Fig 3G). Follow up Tukey’s HSD also exhibited no significance over pairwise mean differences. Analysis of the GFAP activated area, however, revealed significant influence by ApoE4 carriage, F(1,47) =  6.12, p = 0.017 in female group only, while male group showed F(1,29) =  1. 32, p = 0.260. For ApoE4 effect on GFAP activation, the Y group had a significantly higher mean difference of 3808405.96, p =  0.009. Moreover, we observed our first significant interactive effect of ApoE4 and cognitive status within female cohort, F(1,47) =  4.900, p =  0.032. To further explore the interaction of combined effects of ApoE4 and cognitive influence, Tukey’s HSD showed that group of YDem had significantly higher average units than NDem by 5737056.79, p =  0.009, and YDem also exhibited higher level of GFAP activation than NNodem, with a mean difference of 5826021.72, p =  0.006 (Fig 4G). To be noted, YDem group almost had a significant level of GFAP than the YNodem group with a mean difference of 6326227.46, p =  0.051 (Fig 4G). With that being mentioned, astrogliosis is more likely to be involved under the genetic influence of ApoE4, whereas on microglia, it didn’t exhibit the same effect.

Furthermore, we found that the inactivated Iba1 cells (where microglia cells are at resting state) show quite the opposite correlation observed in the activated Iba1 (where microglia cells are at reactive state), and the total combined cells reveal a relatively similar relationship with the activated ones using heatmap (Fig 8A and 8B).

**Fig 5:**
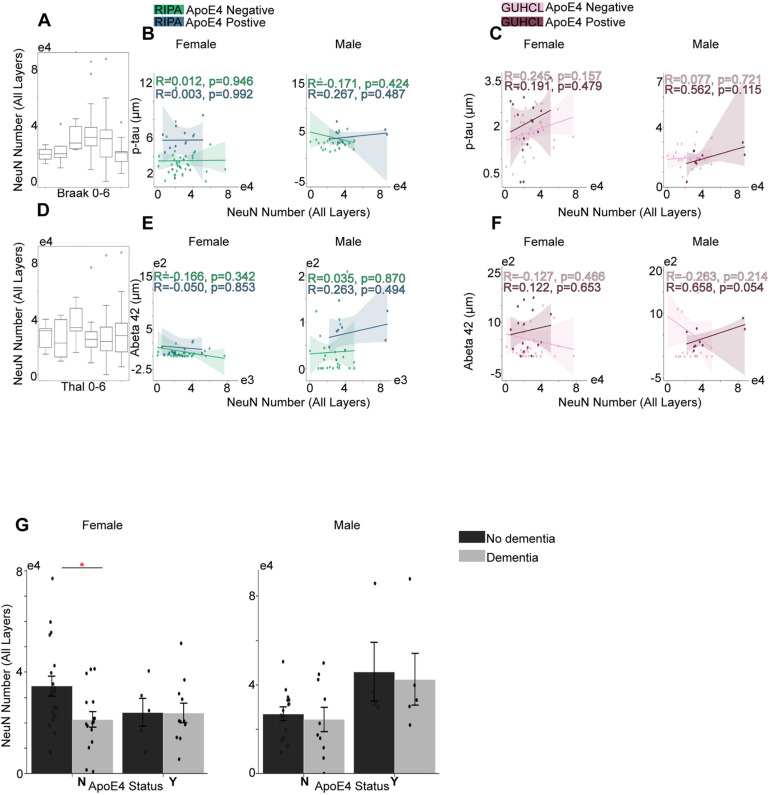
Grey matter NeuN number over the progression of Tau phosphorylation and Aβ aggregation, and their associations with ApoE4 and cognitive status. (A) Total number of NeuN’s progression in correlation with Braak stages. (B) The association between Ripa immunoassay of p-tau level and the total number of NeuN under different sex groups. (C) The association between Guhcl protein precipitation of p-Tau level and the total number of NeuN different sex groups. (D) The total number of NeuN’s progression is in correlation with the Thal stages. (E) The association between Ripa immunoassay of Aβ42 level and the total number of NeuN in different sex groups. (F) The association between Guhcl protein precipitation of Aβ42 level and the total number of NeuN in different sex groups. (G) ApoE4 and Cognitive status influences on NeuN cell aggregation under different sex groups. Pearson’s R correlation was conducted B-C, and E-F. Two-way ANOVA and Tukey’s HSD post-hoc analysis was used for G. Data represent mean ±  SEM. * p <  0.05; **p <  0.01; ***p <  0.001; ****p <  0.0001.

**Fig 6:**
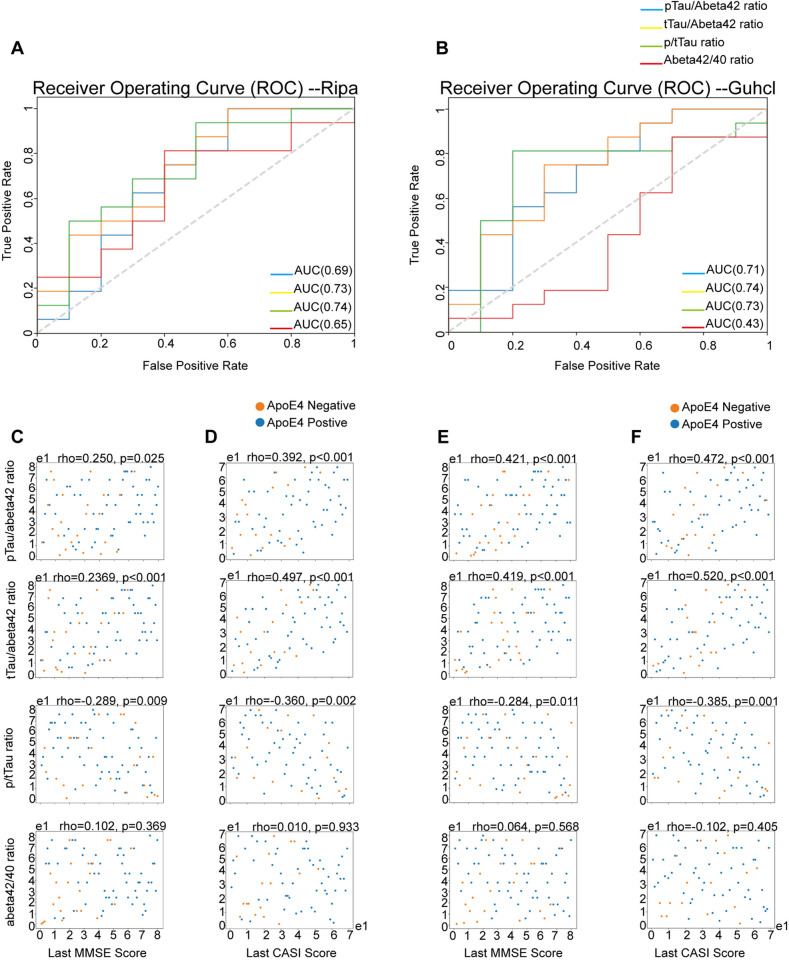
Receiver Operating Characteristics (ROC) curves for different diagnostic ratios in predicting AD dementia. The curves represent the performance of the pTau/Aβ42 ratio (blue), tTau/Aβ42 ratio (green), pTau/tTau ratio (red), and Aβ42/40 ratio (orange). The area under the curve (AUC) for each ratio is indicated in the legend. The grey dashed line represents the no-discrimination line, where the classifier has no predictive ability. (A) ROC curves for the pTau/Aβ42, tTau/Aβ42, pTau/tTau, and Aβ42/40 ratios, measured using the Ripa immunoassay, to predict AD dementia. (B) ROC curves for the pTau/Aβ42, tTau/Aβ42, pTau/tTau, and Aβ42/40 ratios, measured using the Guhcl protein precipitation, to predict AD dementia. (C) Spearman correlation between ranked MMSE scores and pTau/Aβ42, tTau/Aβ42, pTau/tTau, and Aβ42/40 ratios under Ripa immunoassay. (D) Spearman correlation between ranked CASI scores and pTau/Aβ42, tTau/Aβ42, pTau/tTau, and Aβ42/40 ratios under Ripa immunoassay. (E) Spearman correlation between ranked MMSE scores and pTau/Aβ42, tTau/Aβ42, pTau/tTau, and Aβ42/40 ratios under Ripa immunoassay. (F) Spearman correlation between ranked CASI scores and pTau/Aβ42, tTau/Aβ42, pTau/tTau, and Aβ42/40 ratios under Guhcl protein precipitation.

**Fig 7:**
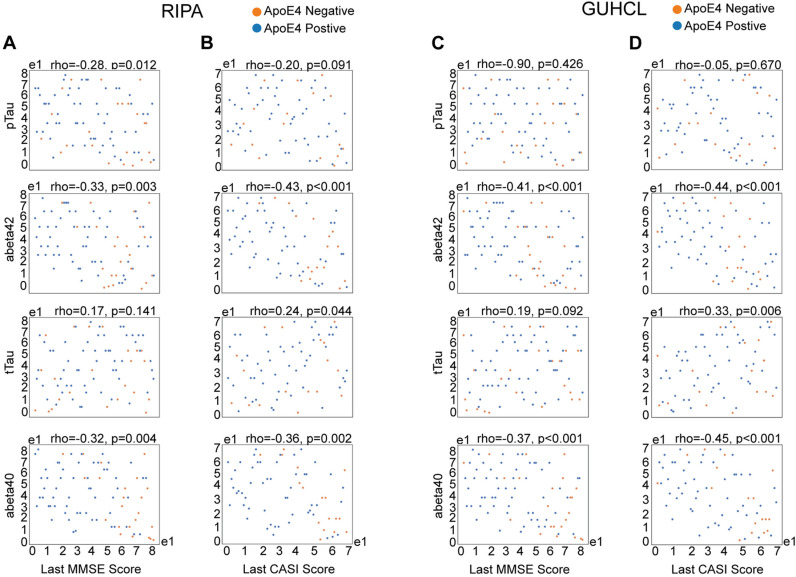
Spearman’s correlation of individual biomarkers and cognitive MMSE and CASI scores under the influence of ApoE4 status. A) Spearman correlation between ranked MMSE scores and pTau, Aβ42, tTau, and Aβ40 under Ripa immunoassay. (B) Spearman correlation between ranked CASI scores and pTau, Aβ42, tTau, and Aβ40 under Ripa immunoassay. (C) Spearman correlation between ranked MMSE scores and pTau, Aβ42, tTau, and Aβ40 under Guhcl protein precipitation. (D) Spearman correlation between ranked CASI scores and pTau, Aβ42, tTau, and Aβ40 under Guhcl protein precipitation.

**Fig 8:**
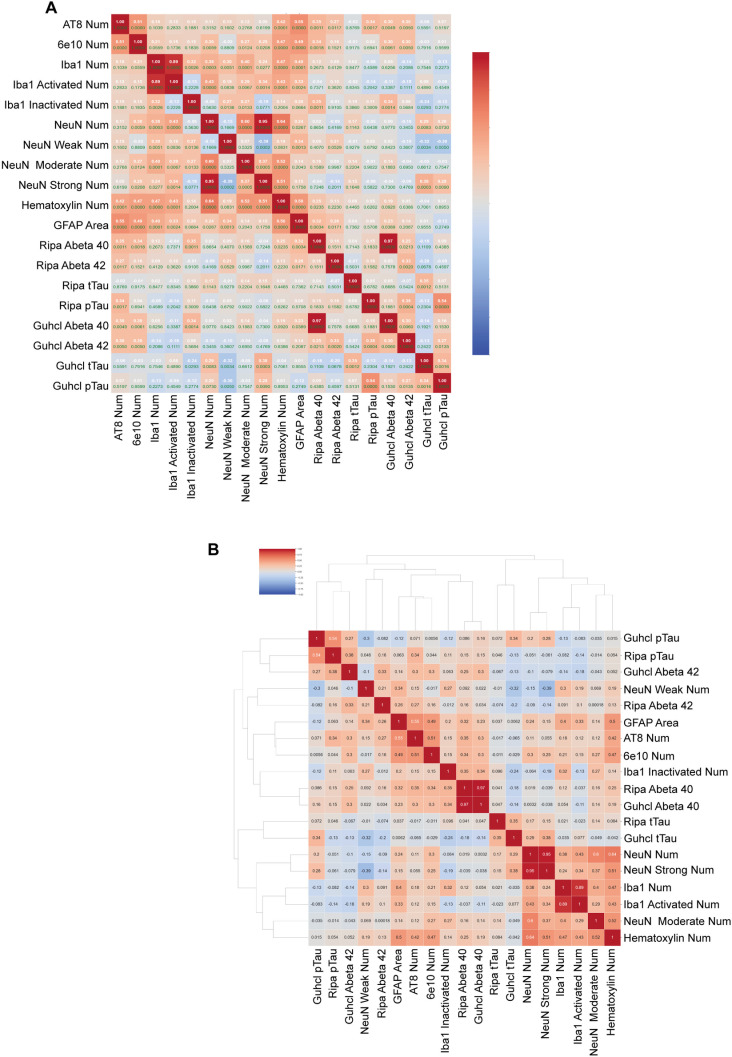
Clustered heatmaps of all pathological biomarkers intercorrelations. Pearson correlation was conducted and labeled with each of the biomarkers represented on the Heatmap. Correlation scale from -1 to 1, that -1 means the least correlated and 1 means the most correlated, indicating by color from blue to red. (A) heatmap of biomarker and antibodies in grey matter with correlation in white font and p value in green labeled in each box. (B) Clustered heatmap of biomarker and antibodies in grey matter with correlation in black font labeled in each box.

### Neuronal loss

Neuronal loss is considered the most common pathology of neurodegenerative disorders, including AD [13,34]. Interestingly, we did not observe any significant correlation of total NeuN in grey matter of the MTG with neither p-tau or Aβ plaques through the progression of AD. Notably, in male ApoE4 positive group (Guhcl-protein precipitation) there was a trend towards positive correlation between NeuN and Aβ42 level, with r =  0.658, p = 0.054 (Fig 5F).

Hematoxylin-stained nuclei shared a similar correlation with total neuronal nuclei on the progression of disease for both tau and Aβ pathology (Fig 8A). Interestingly, not only the number of hematoxylin-stained nuclei but also neuronal nuclei had a positive correlation with the immune-response of glial cells. Hematoxylin-stained nuclei exhibited moderate correlation with activated Iba1 cells r =  0.47, p <  0.001, and r =  0.50, p <  0.001 with GFAP activation. NeuN and Iba1 activation had a r =  0.43, p <  0.001, and with GFAP activation had a r =  0.24, p =  0.027. This suggests a positive relationship between neuronal activity and glial activation. In addition, we also observed that hematoxylin-stained nuclei had a moderate correlation with number of AT8 and 6e10 antibodies, r =  0.42, p <  0.001; r = 0.47, p <  0.001 respectively. However, later heatmap correlation only shows a small correlation with Ripa- Aβ40 level only (Fig 8A).

We further investigate the impact of ApoE4 and cognitive status on NeuN levels. The ANOVA results revealed that ApoE4 status did not have a significant effect on female NeuN levels F (1, 47) =  0.27, p =  0.61. Conversely, cognitive status had a significant effect on NeuN levels F (1, 47) =  5.34, p =  0.03. Following Tukey’s HSD revealed that Nodem group of females had an average NeuN level that was of 9987.25 units higher than Dem group, p =  0.017. Moreover, we also noticed a pairwise difference between the female NDem and NNodem group that the NNodem group had a higher unit with a mean difference of 13207.28, p =  0.045. This suggests that cognitive decline, independent of ApoE4 influence significantly reduced the level of neuronal nuclei in female diseased brain samples. However, we found a significant independent ApoE4 effect but no cognitive effect on male neuronal level, F (1,47) =  6.97, p = 0.013, with the Y group having a higher average unit of 18095.96, p = 0.012 than the N group (Fig 5G). This suggests potential gender differences in ApoE4’s influence on neuronal levels.

### Cognitive performances

The diagnostic performances of the pTau/Aβ42, tTau/Aβ42, pTau/tTau, and Aβ42/40 ratios, measured using both Ripa and Guhcl protein precipitation methods, were evaluated through Receiver Operating Characteristic (ROC) curve analysis for predicting AD dementia [35,36]. Under Ripa measurements, the analysis revealed the following areas under the curve (AUC): pTau/Aβ42 ratio had an AUC of 0.69, indicating moderate accuracy; tTau/Aβ42 ratio demonstrated a higher AUC of 0.73, reflecting better discriminatory power; pTau/tTau ratio achieved an AUC of 0.74, showing the highest level of accuracy among the ratios; while the Aβ42/40 ratio had an AUC of 0.65, indicating the lowest performance among the evaluated ratios (Fig 6A). In comparison, Guhcl measurements revealed pTau/Aβ42 ratio had an AUC of 0.71, the tTau/Aβ42 ratio had an AUC of 0.74, the pTau/tTau ratio had an AUC of 0.73, and the Aβ42/40 ratio exhibited the lowest accuracy with an AUC of 0.43 (Fig 6B). Both measurements suggested the pTau/tTau and tTau/Aβ42 ratios were better at showing discriminatory power compared to the others. To further investigate these findings, Spearman correlation analyses were performed to explore the relationship between the diagnostic ratios and ranked scores from the Mini-Mental State Examination (MMSE) and The Cognitive Abilities Screening Instrument (CASI) assessments [37,38].

We found that all the examined ratios, except for the Aβ42/40 ratio, showed significant Spearman correlations with ranked cognitive scores. Interestingly, the pTau/Aβ42 and tTau/Aβ42 ratios exhibited positive correlations with cognitive scores, which is counterintuitive given that their individual components showed negative correlations with cognitive performance. Specifically, the Ripa-pTau/Aβ42 ratio demonstrated a weak positive correlation with ranked MMSE scores, rho =  0.25, p =  0.025, suggesting a modest association with cognitive performance. In contrast, the Ripa-tTau/ Aβ42 ratio revealed a moderate positive correlation with ranked MMSE scores, rho =  0.37, p <  0.001, indicating a more significant relationship with cognitive scores (Fig 6C and 6D). However, when considering the individual components, the Ripa-pTau, Aβ42, and Aβ40 levels all exhibited negative correlations with MMSE scores: Ripa-pTau had a correlation of rho =  -0.28, p =  0.012; Ripa-Aβ42 had rho =  -0.33, p =  0.003; and Ripa-Aβ40 had rho =  -0.32, p =  0.004 (Fig 7A). The Ripa-tTau level did not show a significant correlation with MMSE scores. Additionally, the Ripa-pTau/tTau ratio displayed a negative correlation with both MMSE and CASI scores (rho =  -0.29, p =  0.009), reflecting an inverse relationship with cognitive performance. In terms of CASI scores, the Ripa-pTau/Aβ42 ratio showed a moderate positive correlation, rho =  0.39, p <  0.001, and the Ripa-tTau/Aβ42 ratio demonstrated an even stronger positive correlation of rho =  0.50, p <  0.001. Conversely, the Ripa-pTau/tTau ratio also exhibited a negative correlation with CASI scores, rho =  -0.36, p =  0.002 (Fig 6E and 6F). For individual biomarkers, the Ripa-Aβ42 level had a negative correlation with CASI scores, rho =  -0.43, p <  0.001, as did the Ripa-Aβ40 level, rho =  -0.36, p =  0.002. The Ripa-tTau level showed a weak positive correlation with CASI scores, rho =  0.24, p =  0.044 (Fig 7B). These results suggest a clear discrepancy between combining ratios and using individual biomarkers in evaluating cognitive scores. In addition, CASI scores might provide a more sensitive measure of cognitive performance in relation to these biomarkers compared to MMSE scores. Similar patterns were observed with the Guhcl diagnostic ratios and ranked cognitive scores.

## Discussion

This study delves into the intricate interplay between the AD pathological biomarkers, focusing on ApoE4 status in correlation with p-tau (AT8) and Aβ (6e10), Hematoxylin-stained nuclei, NeuN, Iba1 and GFAP glia cell activation. Additionally, we seek to unravel the potential associations with the non-pathological markers such as cognitive performances and sex.

Our research revealed significant positive correlation with p-Tau and Aβ42 levels. This corroborated the previous findings on the interconnection between tau-phosphorylation and amyloid plaques [20,28,39]. However, contrary to our expectations, AT8 recognizes Tau phosphorylation primarily in non-ApoE4 carriers. This brought up the question of whether ApoE4 can impede AT8 antibody in recognizing p-tau. In contrast, 6e10 antibody shows specificity in recognizing Aβ plaques in both groups of ApoE4 status [32]. Moreover, Tukey’s HSD test revealed the highest level of AT8 and 6e10 appeared in the YDem group, indicating a possible combined effect of ApoE4 and dementia in exacerbating effect in Tau hyperphosphorylation and Aβ aggregation in AD patients as described in previous studies [11,20,30,33,40]. Indeed, previous studies also corroborated with our current analysis that tauopathy was regarded as a better predictor (compared to Aβ deposits) of cognitive decline, especially in the temporal region of the brain [18,26,41].

Moreover, our study identified a gender-specific, ApoE4-independent effect in aggravating Ripa-pTau level. Tukey’s HSD revealed YDem group with the highest means. Ripa-Aβ42 level wasn’t significantly impacted by neither ApoE4 nor dementia in both sex groups. Guhcl-Aβ42 level was found significantly impacted by dementia only but not ApoE4. This suggests that ApoE4 may specifically enhance the process of Aβ aggregation and plaque formation (6e10 accumulation) rather than affecting the overall production or accumulation of Aβ42. These results highlight the importance of considering ApoE4’s role in modulating Aβ aggregation dynamics, which may have crucial implications for developing therapeutic strategies targeting Aβ pathology in AD.

Furthermore, we found that neither microglia nor astrocytes were reactive over the progression of p-Tau accumulations in both Ripa and Guhcl protein extraction methods. In contrast, both GFAP and Iba1 exhibited strong correlation between their reactivities and Aβ42 levels. One research has demonstrated the reactive astrocytes triggered by activated microglia, which secrete IL-1alpha (IL-1α), IL-1beta (IL-1β), IL-6, tumor necrosis factor-α (TNF-α), and complement component 1q (C1q) [42]. Additionally, another research have identified a feed-forward relationship between the astrocyte stimulation and increased expression of β-site APP-cleaving enzyme (BACE1), APP, and β-secretase [43]. These changes are crucial in the formation of Aβ plaques, suggesting a glial-amyloid-specific pathological pathway that significantly contributes to AD progression.

We found no significant influence of the ApoE4 and dementia on Iba1 activation. However, ApoE4 is associated with aggravation of GFAP activation. In addition, there was an interactive effect of ApoE4 and dementia. Tukey’s HSD showed that YDem group had the highest activation of GFAP area. This suggests that ApoE4 may have a specific modulating effect on astrocytes but not microglia when involving neuroinflammation responses in AD dementia. Research on Iba1 and GFAP activity in AD circumstances is still controversial regarding the role of ApoE4. One study suggested reduced activity of Iba1 and GFAP around the amyloid plaques in APP/PS1 ApoE4 mice under sleep deprivation in brain region of cortex, hippocampus, and thalamus when compared with the APPPS1 apoE3 mice group [44], which shows positive suppression of the Iba1and GFAP activity by ApoE4 gene. However, another study showed that the Iba1 and GFAP’s immunoreactivities are similar between ApoE4 carriers or non-carriers in mice models with minimal amyloid plaques [45]. Moreover, many studies have corroborated positive correlation between the GFAP activation with tauopathy independent of Aβ pathology in AD mouse models. In Thy-Tau22 model representing hyperexcitability of hippocampal astrogliosis and seizure susceptibility with increasing tauopathy [46], and in the PS19 mice model, increased astrocyte activity with major tauopathy along with hyperactive behaviors were observed [47].

Finally, we found no correlation between NeuN and either p-Tau or Aβ42 progression regardless of sex and ApoE4 expressions. However, further ANOVA analysis revealed a gender difference role of ApoE4 in affecting level of NeuN. ApoE4 carriers in male cohort showed higher levels of NeuN in grey matter, which may contradict the results of reduced cortical thickness and hippocampal volume [48,49]. Interestingly, we found moderate to strong positive correlation of the neuronal and hematoxylin-stained nuclei with the Iba1 and GFAP activation. This shows the interconnection between the glial and neuronal activities in grey matter.

To address the issue of preclinical usage of pTau/Aβ42, tTau/ Aβ42, pTau/tTau, and Aβ42/40 ratios in predicting AD and AD dementia, we used ROC curve analysis. We found all the above ratios besides Aβ 42/40 could be a good indicator in differentiating AD dementia and AD non-dementia groups, and the ratios of tTau/ Aβ 42 and pTau/tTau ratios were the two most consistent at showing the strongest discriminatory power. The higher AUC values observed for the tTau/ Aβ 42 and pTau/tTau ratios in both Ripa and Guhcl measurements corroborate earlier studies that have highlighted these ratios as reliable biomarkers in AD diagnostics [50]. Specifically, the tTau/ Aβ42 ratio, which consistently achieved AUC values above 0.70, reinforces its role as a key indicator of Aβ and tau pathology in AD. The pTau/tTau ratio, which showed the highest accuracy with an AUC of 0.74 under Ripa measurements, further underscores its diagnostic relevance, particularly in detecting tau pathology independent of Aβ burden.

We observed different relationships between the level of individual biomarkers and their ratios regarding cognitive performances measured in MMSE and CASI ranked scores. While the individual levels of Ripa-pTau, Aβ 42, and Aβ 40 negatively correlate with cognitive scores, indicating that higher biomarker levels are generally linked with decreased cognitive function, the ratios of pTau/ Aβ 42 and tTau/ Aβ 42 exhibit positive correlations with these scores. This suggests that a higher proportion of tau relative to Aβ might be associated with preserved cognitive function within the cohorts of our study. This apparent paradox in biomarker behavior underscores the complex interplay between tau and amyloid pathologies in AD. A positive correlation for these ratios could imply that while overall increases in these biomarkers indicate disease progression, the rate at which tau pathology increases relative to Aβ might provide unique insights into the severity or stage of the disease and their degree of dementia status. Additionally, the the pTau/tTau ratio findings, which showed a negative correlation with cognitive scores, further underscore the detrimental impact of a higher proportion of phosphorylated tau relative to total tau protein. This ratio provides a direct measure of tau pathology’s severity and its more immediate effects on cognitive decline [26,41].

In summary, our findings indicate the detrimental effect of ApoE4 in accumulating Tau phosphorylation and aggregating Aβ depositions in grey matter. However, we also noticed the inconsistency between Aβ plaque formations and accumulated count of Aβ 42. Moreover, we noticed an Aβ-specific glial reactivities pathology, and ApoE4’s aggravating effects of astrogliosis. Furthermore, the gender differences under ApoE4’s impact was not significant. Notably, we did not notice significant neuronal loss in grey matter compared to previous studies in the hippocampus, which underscores the significance of brain regional differences. Lastly, the contrasting outcomes from our correlation and ROC analyses demonstrate that while the tTau/ Aβ 42 and pTau/tTau ratios are effective at discriminating AD dementia status, they do not necessarily reflect precise levels of cognitive performance. These insights suggest that while individual biomarkers reflect the overall disease burden, their ratios can offer deeper insights into the dynamics between different pathological processes, potentially guiding more targeted diagnostic and therapeutic strategies in AD.

## Method

### Participants

All data were obtained from the Seattle Alzheimer’s Disease Brain Cell Atlas (SEA-AD), provided by the Allen Institute for Brain Science, https://portal.brain-map.org/explore/seattle-alzheimers-disease/seattle-alzheimers-disease-brain-cell-atlas-download. A total of 84 donor brain samples were measured and analyzed by the SEA-AD focused on the brain region of MTG. Within this cohort, 59 individuals were ApoE4-negative (N), and 25 were ApoE4-positive (Y), among which at least one of the alleles is ApoE4. The cohort comprised 51 females and 33 males. Pathological status was recorded using Braak and Thal stages [51–53]. Cognitive status was assessed, with 42 individuals classified as cognitively normal (no dementia), 42 diagnosed with dementia, including one with cognitive impairment but also categorized as dementia by SEA-AD (Table 1). AD stages are categorized based on Alzheimer’s Disease Neuropathological Changes (ADNC) scores: 9 not AD, 12 low AD, 21 intermediate AD, and 42 severe AD. Notably, we included the not AD group to comprehensively study AD progression across all severity levels.

### Data analysis

We utilized Python version 3.11.3 for the analysis and visualization of pathological and cognitive biomarkers, conducted within the Jupyter Notebook interface of the Anaconda distribution. This environment supports interactive computing that seamlessly integrates code execution, visualization, and documentation. For data processing and statistical analysis, we employed key Python libraries. Pandas facilitated data manipulation, NumPy handled numerical computations, and SciPy was used for statistical tests. Visualizations were created using Matplotlib and Seaborn version 13.0, enhancing the clarity and accessibility of our findings. To ensure reproducibility, all Python code used for the analysis is available in a GitHub repository at https://doi.org/10.5281/zenodo.13892186. Supplementary material, including all filtered dataset in assistance for data analysis were also included in the same GitHub repository.

To evaluate the correlations between biomarkers and the influence of genetic and demographic factors, we performed Pearson and Spearman correlation analyses, two-way ANOVA and post-hoc Tukey’s HSD tests. Given the consistent association of ApoE4 and cognitive status across all brain layers, we decided to use “Grey Matter” for each antibody and biomarkers as standard analysis measurements through this study. We separated patient group based on sex status, female and male, and conducted two-way ANOVA and Tukey’s HSD test based on ApoE4 status (negative or positive) and cognitive status (dementia and no dementia) for two-way ANOVA (Table 1). Pearson correlation was conducted between the antibodies and biomarkers, especially with associations of p-Tau and Aβ42 levels. Spearman correlation was used for association between biomarker ratios and ranked cognitive scores, MMSE and CASI.

## Limitations

Our study has some limitations worth noting. Firstly, the sample size is relatively small, with only 84 donors analyzed through autopsy. This hinders the generalizability of our findings to a broader population and may limit the availability of certain data analysis tests due to the smaller cohort. Future studies should aim for larger donor populations to enhance research robustness. Another limitation is the inclusion of outliers in our analysis. Although we chose not to remove them, recognizing their relevance to real human conditions, their presence may influence our overall findings, and the small sample size exacerbates this challenge. Lastly, we didn’t analyze the brain images collected by SEA-AD. Integrating both brain images and biomarkers information in the future could provide a more comprehensive understanding of AD disease pathological progression in specific brain regions.
